# The impact of HER2 discordance on patient prognosis following anti-HER2 neoadjuvant therapy for HER2-positive breast cancer

**DOI:** 10.3389/fonc.2026.1889796

**Published:** 2026-08-04

**Authors:** Yining Ren, Tianlong Su, Zixing Gong, Guolei Li, Hao Feng, Xinyu Feng, Sicheng Zhou, Wei Xing

**Affiliations:** 1 Department of General Surgery, Hebei Provincial Hospital of Chinese Medicine/The First Affiliated Hospital of Hebei University of Chinese Medicine, Shijiazhuang, China; 2 Department of General Surgery, Xilin Gol League Central Hospital, Xilinhot, China; 3 Department of Health Care, Peking Union Medical College Hospital, Chinese Academy of Medical Sciences and Peking Union Medical College, Beijing, China; 4 Department of Thyroid and Breast Surgery, Peking University First Hospital, Beijing, China

**Keywords:** breast cancer, HER2-discordance, HER2-positive, neoadjuvant chemotherapy, prognosis, targeted therapy

## Abstract

**Background:**

Human epidermal growth factor receptor 2 (HER2) discordance between biopsy and residual disease (RD) is common in HER2-positive breast cancer (BC) after anti-HER2 neoadjuvant therapy (NAT). This study investigated the impact of HER2 discordance on oncological outcomes.

**Methods:**

Consecutive HER2-positive BC patients who received anti-HER2 NAT were identified from a prospective database. HER2 status was evaluated for both biopsy specimens and RD before and after NAT. The prognostic implications of HER2 discordance were assessed.

**Results:**

A total of 700 HER2-positive BC patients were enrolled, 267 (38.1%) of whom achieved pathological complete response (pCR) after NAT. For patients with RD after NAT, the HER2 status of RD was HER2 (3+) in 209 (29.9%), HER2 (2+) in 144 (20.6%), and HER2 (1+ or 0) in 80 (11.4%) patients. A total of 217 patients exhibited HER2 changes after NAT, including 183 (26.1%) cases with loss and 34 (4.9%) with gain of HER2 positivity. There was no significant difference (*P*>0.05) in 5-year invasive disease-free survival (IDFS) between patients with different HER2 statuses of RD after NAT (87.3%, 84.2% and 80.9% for no change, loss and gain of HER2 positivity, respectively). Multivariate analysis indicated that cN+ (HR 3.946, 95% CI 1.535-10.148; *P* = 0.004), Miller-Payne grade of 3-4 (HR 0.499, 95% CI 0.278-0.933; *P* = 0.040) and pre-NAT HER2 (3+) (HR 0.479, 95% CI 0.251-0.915; *P* = 0.026) were independent prognostic factors for IDFS in patients without pCR after anti-HER2 NAT.

**Conclusion:**

HER2 discordance after anti-HER2 NAT was observed in a substantial proportion of HER2-positive BC patients. However, neither the change of HER2 nor the specific HER2 status after NAT was associated with the prognosis. Moreover, pre-NAT HER2 status was a robust prognostic factor in HER2-positive BC patients who received anti-HER2 NAT.

## Introduction

The molecular subtype of primary tumors is a crucial determinant of both the treatment strategy and prognosis of breast cancer (BC) patients. In clinical practice, human epidermal growth factor receptor 2 (HER2) is overexpressed in 15-20% of BC cases, often characterized by more aggressive biological behavior, a higher recurrence rate, and worse prognosis after initial treatment (1, 2). With the introduction and continuous development of anti-HER2 targeted drugs such as trastuzumab and pertuzumab, the treatment outcomes of HER2-positive BC have markedly improved, leading to their inclusion in therapeutic indications (3, 4). Neoadjuvant therapy (NAT) in combination with anti-HER2 targeted drugs plays an important role in the initial treatment of HER2-positive BC, as significant tumor regression increases the probability of breast-conserving surgery (BCS) and can significantly improve the prognosis of patients, especially in those who achieve pathological complete response (pCR) (5–7).

Nevertheless, it is well documented that in 15-40% of patients without pCR, the expression of HER2 may change in residual disease (RD) after NAT, which may be related to tumor heterogeneity, NAT-induced clonal substitution, or sampling errors during needle biopsy (8). However, recent studies could not reach a consensus on the impact of HER2‐discordance after NAT on treatment outcomes in HER2-positive BC, with several studies finding that loss of HER2 amplification after NAT is associated with a poor prognosis (8–13), while others concluded that changes of HER2 status in RD after NAT are not associated with significantly different oncological outcomes (14–16). These discordant results may be attributed to four main factors, including: (1) the diversity of NAT regimens and molecular types of the study population, with some studies including patients who did not receive anti-HER2 targeted therapy or HER2-negative patients (8, 9); (2) lack of information on adjuvant treatment strategies (10); (3) inconsistent definitions based on change of HER2 status or loss of HER2 positivity, with some studies including both loss and gain of HER2 positivity (14); as well as (4) differences in the guidelines used for assessment of HER2 status (16). It is therefore important to conduct a deeper investigation of the impact of HER2-discordance on prognosis of HER2-positive BC after NAT, which can provide evidence for the implementation of optimal adjuvant therapy for HER2-positive BC patients. We therefore used the assessment criterion of HER2 status promulgated in the 2018 guidelines of the College of American Pathologists and the American Society of Clinical Oncology (CAP/ASCO) to investigate the effect of HER2 discordance in RD (no change, loss or gain of HER2 positivity) on the prognosis of HER2-positive BC patients receiving anti-HER2 NAT.

## Methods

### Patients and study methodology

This was a retrospective study conducted based on a prospectively collected BC database from Peking University First Hospital and Hebei Provincial Hospital of Traditional Chinese Medicine between January 2015 and June 2023. Women with HER2-positive BC who received anti-HER2 NAT and underwent radical surgery were systematically reviewed and included in this study. The inclusion criteria were as follows: (1) age between 18–75 years; (2) HER2-positive invasive ductal carcinoma confirmed by coarse needle biopsy; (3) NAT with anti-HER2 targeted agents; (4) clinical stage II-III. Patients with distant metastases, less than 6 cycles of NAT, occult BC, bilateral BC, inflammatory BC or a history of other malignancies were excluded from this study. Tissue specimens were obtained before and after NAT from patient who signed an informed consent form, and the study was carried out with the approval of the ethics committees of both institutions (Approval No.: 2023-479-002).

### Treatment strategies

The database contained complete information for each patient, including basic information, clinical characteristics, imaging examinations, pathological results, and survival outcomes. Single-target anti-HER2 NAT (docetaxel/carboplatin/trastuzumab, TCH) was the main regimen before 2020, while dual-target anti-HER2 NAT (docetaxel/carboplatin/trastuzumab/pertuzumab, TCHP) was the main recommendation after 2020. Mastectomy or BCS was performed within 3–4 weeks after completion of the NAT. Patients with pathologically confirmed axillary lymph node invasion before NAT were directly treated with axillary lymph node dissection (ALND) after NAT; otherwise, sentinel lymph node (SLN) biopsy was performed. ALND was performed if the SLN biopsy was positive. Adjuvant targeted therapy, such as trastuzumab, trastuzumab+pertuzumab, and trastuzumab emtansine (TDM-1), was administered to all patients within 4 weeks following surgery, for a total duration of 1 year. Endocrine therapy and/or radiotherapy was administered in accordance with the National Comprehensive Cancer Network guidelines, typically in combination with anti-HER2-targeted therapy (17).

### Pathological assessment

**Figure 1:**
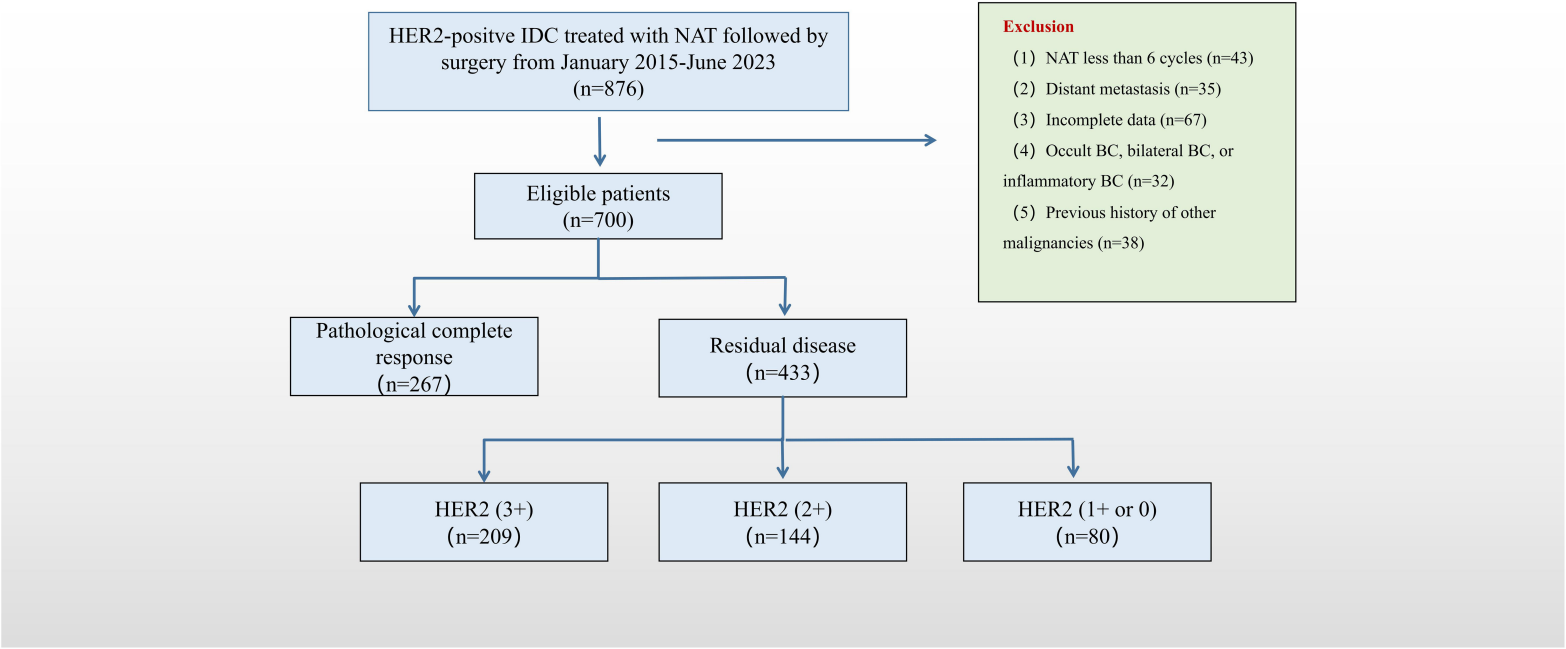
Study flowchart.

Pathological and immunohistochemical (IHC) assays of needle biopsy specimens obtained before NAT and surgical specimens obtained after NAT were performed for each patient. The TNM staging was based on the 8th edition of the American Joint Committee on Cancer (AJCC) staging system (18). The response of the tumors to NAT was evaluated using the Miller-Payne (MP) grading system, while pCR was defined as ypT0/is N0 (19). Estrogen receptor (ER), progesterone receptor (PR), and HER2 status for both pre- and post-NAT specimens was assessed based on the 2018 CAP/ASCO guidelines (20). For patients who were evaluated for IHC before 2018 using the 2013 guidelines, pathologists used the 2018 CAP/ASCO guidelines to re-evaluate the stored tissue specimens. ER/PR status was considered positive when more than 1% of tumor cells were positive for nuclear staining. ER and PR were categorized as HR, with HR-positive indicating positive ER and/or PR and HR-negative indicating negative ER and PR. HER2-positive disease was defined as IHC (3+), or (2+) amplified by fluorescence *in-situ* hybridization (FISH), as well as IHC (0-1) or (2+), while HER2-negative disease was defined by a negative FISH result. FISH was not routinely assessed for patients with HER2 (2+) in RD after NAT. All pathological reports were independently reviewed by two experienced breast pathologists to assess the HER2 scoring using pre-NAT biopsies and postoperative specimens. For discrepant cases with differences of opinion, a third pathologist would be invited to intervene for assessment, and cases where no consensus can be reached will be excluded from this study. In this study, the HER2 status of RD in patients who did not attain pCR after NAT was divided into HER2 (3+), HER2 (2+), and HER2 (1+ or 0), as shown in **Figure 1**. The loss of HER2 positivity was defined as HER2 (3+) changing into HER2 (2+) or HER2 (1+ or 0), or HER2+/FISH (+) changing into HER2 (1+ or 0) after NAT. The gain of HER2 positivity was defined as HER2 (2+)/FISH (+) changing into HER2 (3+) after NAT.

### Statistical analysis

Clinical and pathological variables for patients with residual HER2 (3+), HER2 (2+), and HER2 (1+ or 0) disease were compared using Fisher’s exact test for categorical variables, while the independent samples *t*-test was used for continuous variables. Oncological outcomes based on invasive disease-free survival (IDFS) were estimated from the date of surgery to the first finding of ipsilateral locoregional invasive BC recurrence, contralateral invasive BC recurrence, distant recurrence, or death from any cause. IDFS was calculated using the Kaplan-Meier method, and the log-rank test was used to compare survival outcomes between groups. Univariate and multivariate survival analyses of IDFS were performed using the Cox proportional hazards models. Differences with a *P* value <0.05 were considered statistically significant. Data were analyzed using SPSS 27.0 for Windows (IBM Corp, Armonk, NY, USA).

## Results

A total of 700 HER2-positive patients who received anti-HER2 NAT were included in this study. An overview of the corresponding clinical features and pathological characteristics is shown in **Table 1**. The mean age was 49.3 years, and 332 (47.4%) patients were menopausal at the time of diagnosis. Most of the patients were in clinical stage T1-T2 (n=621, 88.7%). In addition, more than half had lymph node involvement (n=418, 59.7%), while 375 (53.6%) had histological differentiation of grade III. There were 354 (50.6%) patients with positive HR, and most patients had HER2 (3+) (n=583, 83.3%) BC. A total of 316 patients (45.1%) received dual-target anti-HER2 NAT, and mastectomy was the main surgical approach after NAT (n=468, 66.9%). In terms of response to NAT, a total of 591 (83.0%) patients achieved significant tumor regression with MP grade 3-5, 267 (38.1%) of which achieved pCR. Among the patients with RD after NAT, the HER2 status of RD was HER2 (3+) in 209 (29.9%), HER2 (2+) in 144 (20.6%), and HER2 (1+ or 0) in 80 (11.4%) patients. In addition, loss of HER2 positivity was observed in 183 (26.1%) patients and gain of HER2 positivity in 34 (4.9%). In terms of adjuvant therapy, 28 (4.0%) patients received TDM-1 targeted therapy and 441 (63.0%) received radiation therapy.

**Table 1:** Baseline characteristics of HER2-positive breast cancer patients receiving anti-HER2 NAT.

Variables	Overall (n=700)
Age (years, mean ± SD)	49.3 ± 9.8
ECOG	
0-1	462 (66.0)
2	238 (34.0)
Menstrual state	
Yes	332 (47.4)
No	368 (52.6)
cT stage	
cT1-T2	621 (88.7)
cT3-T4	79 (11.3)
cN stage	
Negative	282 (40.3)
Positive	418 (59.7)
Pre-NAT tumor grade	
I-II	325 (46.4)
III	375 (53.6)
Pre-NAT HR status	
HR positive	354 (50.6)
HR negative	346 (49.4)
Pre-NAT Ki67 expression	
<20%	44 (6.3)
≥20%	656 (93.7)
Pre-NAT HER2 status	
HER2 3+	583 (83.3)
HER2 2+/FISH-positive	117 (16.7)
NAT regimen	
TCHP	316 (45.1)
TCH	384 (54.9)
Surgical procedure	
Mastectomy	468 (66.9)
BCS	232 (33.1)
Miller-Payne grade	
Grade 1-2	119 (17.0)
Grade 3-5	591 (83.0)
pCR	
Yes	267 (38.1)
No	433 (61.9)
Post-NAT HER2 status	
pCR	267 (38.1)
HER2 (3+)	209 (29.9)
HER2 (2+)	144 (20.6)
HER2 (1+or 0)	80 (11.4)
Change of HER2	
pCR	267 (38.1)
No change	216 (30.9)
Gain of HER2-positivity	34 (4.9)
Loss of HER2-positivity	183 (26.1)
Adjuvant targeted therapy	
Trastuzumab or Trastuzumab+pertuzumab	672 (96.0)
Trastuzumab emtansine (TDM-1)	28 (4.0)
Adjuvant radiation	
Yes	441 (63.0)
No	259 (37.0)

ECOG, Eastern Cooperative Oncology Group; HR, hormone receptors; BCS, breast conserving surgery; HER2, human epidermal growth factor receptor 2; NAT, neoadjuvant therapy; pCR, pathological complete response.

### Clinicopathological characteristics associated with post-NAT HER2 status

The clinical and pathological characteristic associated with post-NAT HER2 status are listed in **Table 2**. Among patients with HER2 (3+) in RD, the proportion of HR-positive patients was significantly lower than that of HER2 (2+) and HER2 (1+ or 0) (*P* = 0.046). In addition, patients with HER2(3+) before NAT were significantly more likely to have RD with HER2(3+) after NAT rather than HER2(2+) or HER2(1+ or 0) (*P* = 0.016). There were no statistically significant differences between the groups in terms of age, Eastern Cooperative Oncology Group score, cT stage, cN stage, tumor grade, Ki-67 index, NAT regimen, surgical approach, MP grade, adjuvant targeted therapy or adjuvant radiotherapy (*P*>0.05 in all cases).

**Table 2:** Baseline characteristics associated with post-NAT HER2 status.

Variables	HER2 (3+) (n=209)	HER2 (2+) (n=144)	HER2 (1+or 0) (n=80)	*P*
Age (years, mean ± SD)	49.2 ± 9.7	49.3 ± 9.9	48.8 ± 10.9	0.094
ECOG				0.714
0-1	133 (63.6)	93 (64.6)	55 (68.8)	
2	76 (36.7)	51 (35.4)	25 (31.2)	
Menstrual state				0.378
Yes	92 (44.0)	72 (50.0)	33 (41.3)	
No	117 (56.0)	72 (50.0)	47 (58.7)	
cT stage				0.900
cT1-T2	184 (88.0)	127 (88.2)	69 (86.3)	
cT3-T4	25 (12.0)	17 (11.8)	11 (13.7)	
cN stage				0.066
Negative	70 (33.5)	62 (43.1)	37 (46.2)	
Positive	139 (66.5)	82 (56.9)	43 (53.8)	
Pre-NAT tumor grade				0.681
I-II	106 (50.7)	70 (48.6)	36 (45.0)	
III	103 (49.3)	74 (51.4)	44 (55.0)	
Pre-NAT HR status				**0.046**
HR positive	108 (51.7)	88 (61.1)	53 (66.3)	
HR negative	101 (48.3)	56 (38.9)	27 (33.7)	
Pre-NAT Ki67 expression				0.091
<20%	19 (9.1)	5 (3.5)	4 (5.0)	
≥20%	190 (90.9)	139 (96.5)	76 (95.0)	
Pre-NAT HER2 status				**0.016**
HER2 3+	175 (83.7)	103 (71.5)	59 (73.8)	
HER2 2+/FISH-positive	34 (16.3)	41 (28.5)	21 (26.2)	
NAT regimen				0.309
TCHP	68 (32.5)	55 (38.2)	33 (41.3)	
TCH	141 (77.5)	89 (61.8)	47 (58.7)	
Surgical procedure				0.545
Mastectomy	144 (68.9)	102 (70.8)	51 (63.8)	
BCS	65 (31.1)	42 (29.2)	29 (26.2)	
Miller-Payne grade				0.059
Grade 1-2	48 (23.0)	49 (34.0)	22 (27.5)	
Grade 3-4	161 (77.0)	95 (66.0)	68 (72.5)	
Adjuvant targeted therapy				0.209
Trastuzumab or Trastuzumab+pertuzumab	200 (95.7)	132 (91.7)	73 (91.3)	
Trastuzumab emtansine (TDM-1)	9 (4.2)	12 (8.3)	7 (8.7)	
Adjuvant radiation				0.569
Yes	134 (64.1)	88 (61.1)	46 (57.5)	
No	75 (34.0)	56 (38.9)	34 (42.5)	

ECOG, Eastern Cooperative Oncology Group; HR, hormone receptors; BCS, breast conserving surgery; HER2, human epidermal growth factor receptor 2; NAT, neoadjuvant therapy.

Bold fonts indicate statistically significant differences in the P values.

### Survival analysis

**Figure 2:**
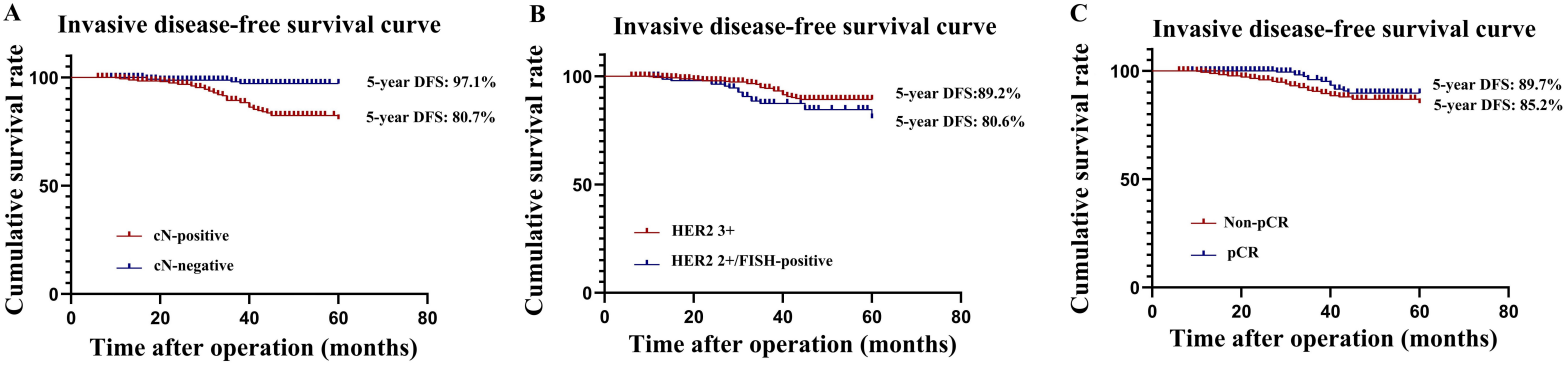
IDFS of HER2-positive BC patients following anti-HER2 NAT; **(A)** comparison of IDFS between patients with cN0 and cN+; **(B)** comparison of IDFS between patients with HER2(3+) and HER2 (2+)/FISH(-) BC; **(C)** comparison of IDFS between patients with pCR and non-pCR.

A total of 26 patients were lost to follow-up, with a median follow-up period of 52 months (range: 12 to 102 months). During the follow-up period, a total of 50 cases of IDFS events occurred. **Table 3** shows the results of the uni- and multivariate analyses of IDFS among 700 patients with HER2-positive BC who underwent radical surgery after NAT. The 5-year IDFS of patients with cN0 and pre-NAT HER2 (3+) was significantly longer than in those with cN+ (97.1% vs. 80.7%, *P* < 0.001) and pre-NAT HER2 (2+)/FISH(+) (89.2% vs. 80.6%, *P* = 0.041) after targeted NAT (**Figures 2A, B**). In addition, the 5-year IDFS was significantly longer in patients who achieved pCR after NAT than in those who did not (89.7% vs. 85.2%, *P* = 0.04; **Figure 2C**). In multivariate analysis, cN+ (HR 4.888, 95% CI 2.105-11.392; *P* < 0.001), pre-NAT HER2 (3+) (HR 0.573, 95% CI 0.302-0.934; *P* = 0.043), and pCR (HR 0.531, 95% CI 0.262-0.911; *P* = 0.037) were independent prognostic factors for IDFS.

**Table 3:** Univariable and multivariable analyses associated with invasive disease-free survival for HER2-positive breast cancer patients following anti-HER2 NAT.

Variables	Invasive disease-free survival			
	Univariate analysis		Multivariate analysis	
	HR (95%CI)	*P*	HR (95%CI)	*P*
Age (years, mean ± SD)	0.982 (0.956-1.010)	0.203		
Menstrual state		0.903		
No	Reference			
Yes	1.035 (0.594-1.803)			
cT stage		0.964		
cT1-T2	Reference			
cT3-T4	0.980 (0.418-2.302)			
cN stage		**<0.001**		**<0.001**
Negative	Reference		Reference	
Positive	6.020 (2.390-15.168)		4.888 (2.105-11.392)	
Pre-NAT tumor grade		0.579		
I-II	Reference			
III	0.854 (0.490-1.490)			
Pre-NAT HR status		0.930		
HR negative	Reference			
HR positive	0.975 (0.558-1.703)			
Pre-NAT Ki67 expression		0.176		
<20%	Reference			
≥20%	0.547 (0.380-1.921)			
Pre-NAT HER2 status		**0.041**		**0.043**
HER2 2+/FISH-positive	Reference		Reference	
HER2 3+	0.550 (0.319-0.941)		0.573 (0.302-0.934)	
NAT regimen		0.083		
TCH	Reference			
TCHP	0.628 (0.385-1.380)			
Surgery		0.155		
Mastectomy	Reference			
BCS	2.413 (0.872-7.921)			
pCR		**0.045**		**0.037**
No	Reference		Reference	
Yes	0.538 (0.286-0.943)		0.531 (0.262-0.911)	
Adjuvant targeted therapy		0.302		
Trastuzumab or Trastuzumab+pertuzumab	Reference			
Trastuzumab emtansine (TDM-1)	2.183 (0.781-9.332)			
Adjuvant radiation		0.284		
Yes	Reference			
No	2.972 (0.833-8.852)			

ECOG, Eastern Cooperative Oncology Group; HR, hormone receptors; BCS, breast conserving surgery; HER2, human epidermal growth factor receptor 2; NAT, neoadjuvant therapy; pCR, pathological complete response; Bold fonts indicate statistically significant differences in the P values.

**Figure 3:**
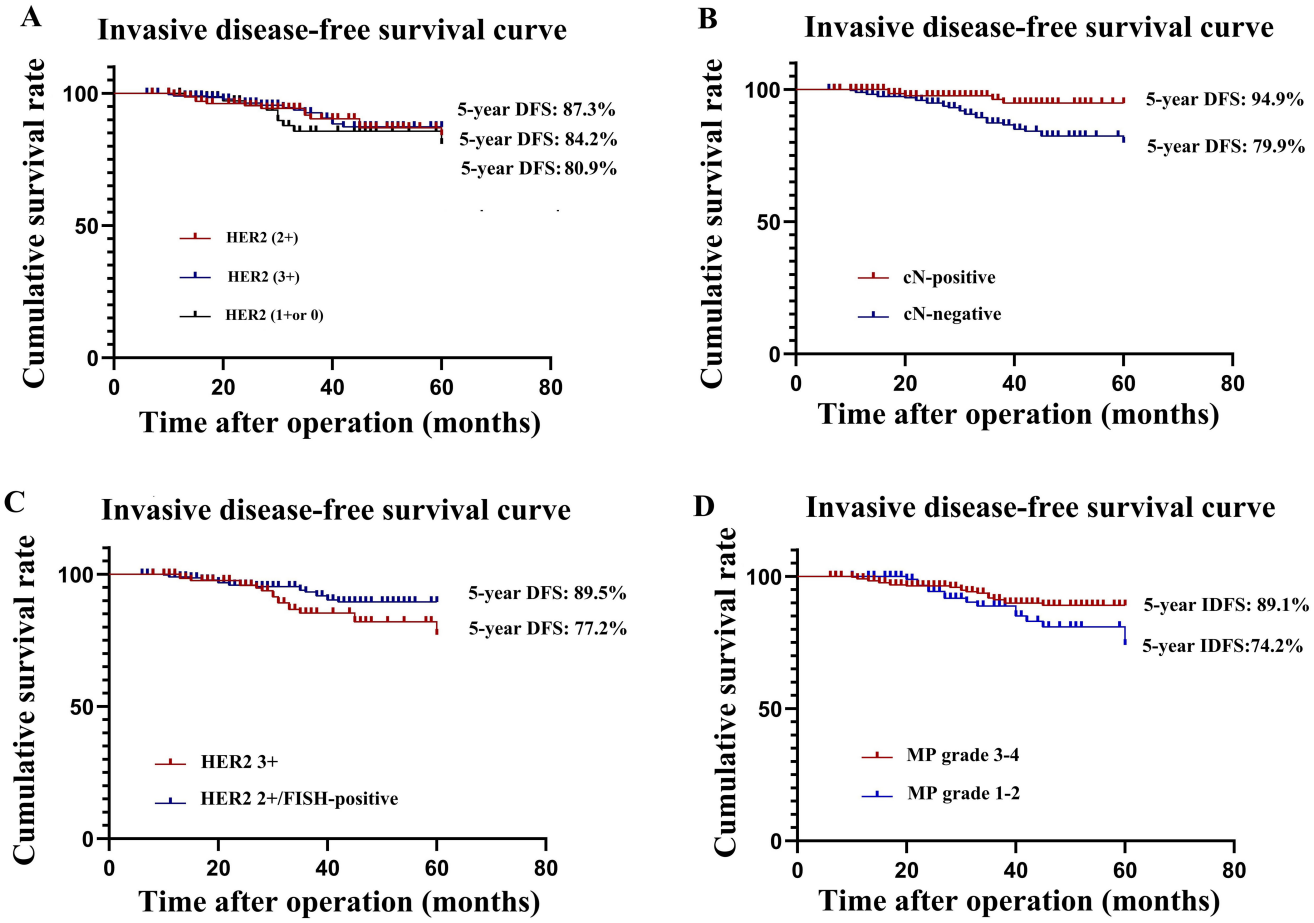
IDFS of HER2-positive BC patients without pCR following anti-HER2 NAT; **(A)** comparison of IDFS between patients with different post-NAT HER2 statuses; **(B)** comparison of IDFS between patients with cN0 and cN+; **(C)** comparison of IDFS between patients with HER2(3+) and HER2 (2+)/FISH(-) BC; **(D)** comparison of IDFS between patients with MP grade of 3–4 and grade of 1-2.

In addition, univariate and multivariate subgroup analyses were performed for the 433 patients who did not achieve pCR following NAT (**Table 4**). Among these populations, a total of 37 IDFS events occurred during the follow-up period. It should be noted that there was no significant difference (*P*>0.05) in 5-year IDFS between patients with different HER2 statuses of RD after NAT (87.3%, 84.2%, and 80.9%; **Figure 3A**). Moreover, the 5-year IDFS of patients with cN0 (94.9% vs. 79.9%, *P* = 0.006), MP grade of 3-4 (89.1% vs. 74.2%, *P* = 0.032), and pre-NAT HER2 (3+) (89.5% vs. 77.2%, *P* = 0.047) was significantly better than that of patients with cN+, MP grade of 1–2 and pre-NAT HER2(2+)/FISH(-), respectively (**Figures 3B–D**). In the multivariate Cox regression analysis, three variables emerged as independent prognostic factors, namely cN+ (HR 3.946, 95% CI 1.535-10.148; *P* = 0.004), MP grade of 3-4 (HR 0.499, 95% CI 0.278-0.933; *P* = 0.040) and pre-NAT HER2 (3+) (HR 0.479, 95% CI 0.251-0.915; *P* = 0.026).

**Table 4:** Univariable and multivariable analyses associated with invasive disease-free survival for HER2-positive breast cancer patients without pCR following anti-HER2 NAT.

Variables	Invasive disease-free survival			
	Univariate analysis		Multivariate analysis	
	HR (95%CI)	*P*	HR (95%CI)	*P*
Age (years, mean ± SD)	0.964 (0.935-1.005)	0.088		
Menstrual state		0.136		
No	Reference			
Yes	0.592 (0.297-1.179)			
cT stage		0.932		
cT1-T2	Reference			
cT3-T4	0.880 (0.417-2.398)			
cN stage		**0.006**		**0.004**
Negative	Reference		Reference	
Positive	3.706 (1.444-9.512)		3.946 (1.535-10.148)	
Pre-NAT tumor grade		0.358		
I-II	Reference			
III	0.738 (0.386-1.410)			
Pre-NAT HR status		0.965		
HR negative	Reference			
HR positive	1.015 (0.530-1.943)			
Pre-NAT Ki67 expression		0.304		
<20%	Reference			
≥20%	0.538 (0.165-1.754)			
Pre-NAT HER2 status		**0.047**		**0.026**
HER2 2+/FISH-positive	Reference		Reference	
HER2 3+	0.523 (0.274-0.993)		0.479 (0.251-0.915)	
NAT regimen		0.133		
TCH	Reference			
TCHP	0.628 (0.385-1.508)			
Surgery		0.215		
Mastectomy	Reference			
BCS	0.733 (0.441-2.701)			
Miller-Payne grade		**0.032**		**0.040**
Grade 1-2	Reference		Reference	
Grade 3-4	0.504 (0.253-0.908)		0.499 (0.278-0.933)	
Post-NAT HER2 status				
HER2 (3+)	Reference			
HER2 (2+)	1.465 (0.641-3.349)	0.365		
HER2 (1+or 0)	1.157 (0.550-2.433)	0.700		
Change of HER2 status				
No change	Reference			
Elevation of HER2	1.432 (0.644-2.291)	0.682		
Loss of HER2	1.893 (0.684-3.892)	0.393		
Adjuvant targeted therapy		0.193		
Trastuzumab or Trastuzumab+pertuzumab	Reference			
Trastuzumab emtansine (TDM-1)	2.494 (0.804-7.922)			
Adjuvant radiation		0.344		
Yes	Reference			
No	2.502 (0.704-8.902)			

ECOG, Eastern Cooperative Oncology Group; HR, hormone receptors; BCS, breast conserving surgery; HER2, human epidermal growth factor receptor 2; NAT, neoadjuvant therapy. Bold fonts indicate statistically significant differences in the P values.

To further evaluate the prognostic impact of HER2 discordance, subgroup analyses was conducted (**Table 5**). The results indicated that the patients were grouped based on pre-NAT HR status, pre-NAT HER2 status, NAT regimen, and postoperative adjuvant therapy. Subgroup analysis showed that there was no significant relationship between post-NAT HER2 status and invasive disease-free survival (all *P*>0.05).

**Table 5:** The impact of HER2 status after NAT on invasive disease-free survival in subgroup analyses.

Variables	Invasive disease-free survival	
	Univariate analysis	
	HR (95%CI)	*P*
Pre-NAT HR-positive cohort		
Post-NAT HER2 (3+)	Reference	
Post-NAT HER2 (2+)	1.998 (0.699-5.712)	0.196
Post-NAT HER2 (1+or 0)	1.047 (0.351-3.120)	0.934
Pre-NAT HR-negative cohort		
Post-NAT HER2 (3+)	Reference	
Post-NAT HER2 (2+)	0.812 (0.172-3.828)	0.793
Post-NAT HER2 (1+or 0)	1.324 (0.479-3.654)	0.588
Pre-NAT HER2 3+ cohort		
Post-NAT HER2 (3+)	Reference	
Post-NAT HER2 (2+)	0.753 (0.098-5.792)	0.785
Post-NAT HER2 (1+or 0)	0.778 (0.292-2.074)	0.616
Pre-NAT HER2 2+/FISH-positive cohort		
Post-NAT HER2 (3+)		
Post-NAT HER2 (2+)	1.506 (0.399-5.678)	0.545
Post-NAT HER2 (1+or 0)	1.966 (0.508-7.609)	0.328
NAT regimen (TCHP)		
Post-NAT HER2 (3+)	Reference	
Post-NAT HER2 (2+)	0.631 (0.170-2.346)	0.492
Post-NAT HER2 (1+or 0)	0.571 (0.191-1.704)	0.315
NAT regimen (TCH)		
Post-NAT HER2 (3+)	Reference	
Post-NAT HER2 (2+)	2.572 (0.829-7.980)	0.102
Post-NAT HER2 (1+or 0)	1.895 (0.657-5.464)	0.237
Adjuvant targeted therapy (Trastuzumab emtansine)		
Post-NAT HER2 (3+)	Reference	
Post-NAT HER2 (2+)	1.452 (0.791-7.911)	*0.502*
Post-NAT HER2 (1+or 0)	1.842 (0.721-6.921)	*0.366*
Adjuvant targeted therapy (Trastuzumab or Trastuzumab+pertuzumab)		
Post-NAT HER2 (3+)		
Post-NAT HER2 (2+)	1.429 (0.605-5.601)	*0.582*
Post-NAT HER2 (1+or 0)	1.524 (0.590-5.592)	*0.602*

HR, hormone receptors; HER2, human epidermal growth factor receptor 2; NAT, neoadjuvant therapy.

## Discussion

This study showed that in patients who did not achieve pCR after anti-HER2 NAT, the incidence of HER2 discordance in RD was 50.1% (217/433), with the majority representing loss of HER2 positivity (84.3%, 183/217). By contrast, gain of HER2 positivity was rare (15.7%, 34/217). The incidence of HER2 discordance was reported inconsistently in the literature (8–16), which may be attributed to differences in objective factors such as the pre-NAT molecular typing of the included population, NAT regimen, and interpretation criteria for assessing HER2 status. In view of the high clinical incidence of HER2 status change in RD after NAT, it is crucial to explore the impact of HER2 discordance on the prognosis, since active adjustment of adjuvant therapy strategies may enable patients with altered HER2 status to achieve better survival benefits. However, the results of this study indicate that neither the change of HER2 status nor the specific HER2 status after NAT is significantly correlated with the prognosis of patients with HER2-positive BC.

The molecular mechanism driving HER2 discordance after NAT remains unclear, but may be related to treatment-induced clonal selection or intrinsic heterogeneity of HER2 expression in tumors (21). Previous studies on the oncological significance of HER2-discordance following NAT have came to different conclusions. Wang et al. (10) found evidence that the loss of HER2 positivity after NAT could identify non-pCR patients with high risk of disease relapse, but almost one-third of the patients included in their study received NAT without targeted drugs. Although Yoshida et al. (14) pointed out that changes of the HER2 status after NAT did not affect the prognosis, the study included patients who were either HER2-positive or -negative before NAT, and the definition of HER2 change included both gain and loss of HER2 positivity, without a specific analysis of HER2 status. A multicenter study conducted by Wetzel et al. (15) demonstrated that the loss of HER2 positivity following NAT was not associated with a worse prognosis. It is important to note that the criteria for diagnosing HER2 status were updated during the study period, and there are significant differences between HER2 status assessments based on different guidelines (21). Uczkowski et al. (16) investigated the significance of HER2-discordance in RD assessed based on 2018 CAP/ASCO guidelines, but only 71 patients were included in the study. In our study, all 700 participants were HER2-positive patients receiving anti-HER2 NAT, and the HER2 evaluation criteria were decided uniformly based on the 2018 CAP/ASCO guidelines. Based on the above inclusion criteria, our investigation of the association of post-NAT HER2 status with oncological outcomes demonstrated that neither the specific status of HER2 nor the change of HER2 status significantly affected the prognosis of HER2-positive patients after NAT.

It is well known that pCR is a crucial indicator for evaluating the response to NAT, indicating a better prognosis in patients with HER2-positive BC following NAC (6). Although both HER2 (3+) and HER2(2+)/FISH-positive subtypes were defined as HER2-positive, HER2 (3+) exhibited a significantly stronger response to NAT than the HER2(2+)/FISH-positive subtype, which may be related to HER2 surface protein levels as well as distinct histological grades or HR positivity rates between the two subtypes. Previous literature (22–24) indicates that HER2 (3+) is associated with a higher pCR rate and better prognosis after anti-HER2 NAT. Similarly, the multivariate analysis in this study showed that the independent prognostic factors influencing IDFS after anti-HER2 NAT in HER2-positive patients were clinical stage, pre-NAT HER2 status, and pCR. In addition, this study further explored the prognostic factors of patients who did not achieve pCR after anti-HER2 NAT, and also found that cN stage, pre-NAT HER2 status, and MP grade were independent prognostic factors affecting IDFS. Notably, this also indirectly indicated that patients with HER2(2+)/FISH-positive BC had low sensitivity to anti-HER2 NAT, less tumor regression, and a low MP grade, resulting in poor prognosis. Therefore, in the era of targeted therapy for HER2-positive BC, the treatment of patients with HER2(2+)/FISH-positive BC is still difficult and needs more attention.

Although the study concludes that HER2 discordance is not associated with prognosis, and TDM-1 remains the recommended regimen for tumor residue after NAT in HER2-positive BC, regardless of HER2 status (25), the reassessment of HER2 status in RD after NAT is still should be routinely recommended. First, HER2 status exhibits spatiotemporal heterogeneity. Nearly 30% of early-stage BC patients will experience changes in HER2 status after NAT, and the changes are bidirectional (26). Not only may HER2-positive cases turn negative, but originally HER2-negative cases also have the possibility of turning positive (27). In addition, the determination of HER2 status can provide a therapeutic basis for subsequent precise adjuvant treatment. Currently, for patients with RD who relapse after TDM-1, HER2 status provides a direct basis for subsequent targeted drug selection. For example, for HER2-positive or HER2-low recurrent or metastatic BC patients, TDX-D can be a more effective option (28, 29). In the current numerous clinical trials targeting residual lesions, the selection of adjuvant treatment strategies remains a key focus for future exploration. The inclusion criteria for these studies all rely on the latest HER2 status after NAT, clarifying the post-NAT HER2 status can provide a basis for patients to participate in more appropriate clinical trials.

In spite of the generally robust findings, there are also some weaknesses that must be considered when interpreting the results of this study. Data analysis in this study was based on a prospective database, but the nature of the study was retrospective, and information on many important factors, such as the time interval between NAT and surgery, radiotherapy dose, duration of endocrine therapy, etc., could not be accurately obtained. In addition, HER2-positive BC has entered the era of dual-target NAT with nearly double the pCR rate compared to single-target NAT (6). However, as the research period of this study was long, nearly half of the patients still received single-target NAT, which may have a certain impact on the results. Moreover, the study period spans from 2015 to 2023, during which treatment strategies for HER2-positive breast cancer changed considerably. Dual-targeted therapy has become widespread in NAT phases, postoperative adjuvant regimens have been altered, and antibody-drug conjugates have been innovated. These treatment differences may have influenced long-term outcomes and potentially confounded the prognostic analyses. Therefore, we conducted subgroup analyses based on factors such as HR status, neoadjuvant treatment regimens, and preoperative HER2 expression levels, with the aim of enhancing the scientific nature and clinical significance of the study.

In addition, a major limitation of the study is the assessment of post-treatment HER2 status. Patients with residual HER2 (2+) disease did not routinely undergo FISH testing after NAT. TDM-1 remains an option for HER2-positive BC patients with RD after NAT, regardless of HER2 status, whether or not FISH testing is used for HER2 (2+) patients does not determine subsequent medication choices. However, the aim of this study was to explore the impact of HER2 status on prognosis after NAT. Patients with undetermined HER2 status could not be classified into detailed groups, the true HER2 status of a substantial proportion of residual tumors remains uncertain, thus having a significant impact on the results of this study. In addition, with the advancement of antibody-drug conjugates, the precise classification of HER2 status plays a crucial role in the subsequent drug selection for patients. Therefore, prospective studies are still needed in the future to accurately assess HER2 status, clarify the impact of HER2 status, especially HER2 (2+), on prognosis, and enable precise treatment for different types of patients.

In conclusion, our study showed that HER2 discordance is observed in a substantial proportion of HER2-positive BC cases after anti-HER2 NAT. However, neither the change nor the specific HER2 status after NAT was associated with the prognosis of HER2-positive BC patients. Moreover, pre-NAT HER2 status was identified as a crucial factor affecting the prognosis of HER2-positive BC patients who received anti-HER2 NAT.

## Data Availability

The original contributions presented in the study are included in the article/supplementary material. Further inquiries can be directed to the corresponding author/s.
